# Digital divide, e-government and trust in public service: The key role of education

**DOI:** 10.3389/fsoc.2023.1140416

**Published:** 2023-02-21

**Authors:** Diego Mesa

**Affiliations:** Department of Sociology, Università Cattolica del Sacro Cuore, Milan, Italy

**Keywords:** education, digital divide, trust in public service, digitization, Italy

## Abstract

The Italian public administration (PA) has always had problems with slowness and inefficiency. In 2021, the Italian government made a massive investment in digitizing PA as part of an extraordinary recovery plan, with more than 200 billion euro to revitalize the country. This paper aims to investigate how educational inequalities affect the relationship between Italian citizens and PA in this phase of the digital transition. The study is based on a web survey conducted in March and April 2022 among a national sample of 3,000 citizens aged 18–64. The data shows that more than three-quarters of respondents have already used a public service at least once *via* an online channel. Few are aware of the reform plan, however, and more than one-third fear that the digitization of public services will make things worse for citizens. Through a regression analysis, the study confirms the centrality of the influence of education on the use of digital public services compared to the other spatial and social variables considered. Trust in PA is also correlated with education and employment status and is higher among those who have used digital public services. The survey thus highlights that the educational and cultural dimension is a crucial aspect as a lever to counter the digital divide and promote digital citizenship rights. It reveals the need to activate facilitation and accompaniment processes for citizens with less digital skills and experience who risk being excluded or penalized by the new arrangement and having their distrust toward the PA and state exacerbated.

## 1. Introduction

This paper examines the relationship between citizens and public services in the Italian context and explores how digital technologies are changing the nature of this relationship. The pervasive diffusion of digital technologies has been one of the most significant factors (both economic, social and cultural) in the morphogenesis of late modern social systems (Castells, 2011; Archer, 2014; Lupton, 2015). On the technological level, observers have spoken of a “new wave of technological innovation” (Perez, 2015); on the economic-productive level, the shift has been described as a “fourth industrial revolution” (Schwab, 2017) or “digital capitalism” (Pace, 2018). With terms such as “digital era,” “data revolution” (Kitchin, 2014), “platform society” (Van Dijck et al., 2018), and “digital modernity” (Lyon, 2017), scholars have sought to emphasize the epochal change taking place in recent decades due to digital technologies, stressing that this shift represents a global break with previous social arrangements. The COVID-19 pandemic has further accelerated these transformative processes by making digital technologies increasingly indispensable in sectors such as work, education, entertainment, shopping, and access to services.

The digital transformation is also producing profound changes in the public administration sector, changes that have repercussions on relations with citizens and businesses (Fang, 2022). The term “e-government” refers to the multiple aspects of this transformation: the use of internet technology to support government operations, provide government services (e-service), and engage citizens (e-democracy) (Meier and Terán, 2012). According to West (2005), the development of e-government systems takes place in progressive stages: (1) the billboard stage, (2) the partial service-delivery stage, (3) the portal stage with fully executable and integrated service delivery, and (4) interactive democracy with public outreach and structures to ensure accountability.

The implementation of public systems through ICT has increasingly become a strategic objective of governments; at the same time, relatives levels of digitization have become established as a benchmark for assessing the development and functioning of states (OECD, 2019, 2020; European Commission, 2022a). In line with this perspective, the OECD (2016) report target-specific public-sector areas in which governments need to adopt new strategies to ensure they keep pace with societal development: healthcare and social care, education, and protection services.

### 1.1. Digital citizenship and public services digitization in the EU and Italy

The transformations currently taking place entail not only an improvement in the efficiency and competitiveness of state apparatuses but also a reshaping of the relationship between states and citizens in a public space that expands into the virtual sphere. The theoretical frame used here views citizenship as a dynamic and open-ended process that develops over time, alongside the evolution of states and the conditions of social life (Marshall, 1950). In this sense, “digital citizenship” can be understood as referring to the set of rights, duties, and opportunities that take shape in the context of specific state organizations that develop digital infrastructure and services (Masucci, 2019).

The European Union has expressly tied its vision for the continent's technological development to an ideal of protecting and promoting digital citizenship. As established in the “European Declaration on Digital Rights and Principles for the European Commission” (2022b), the union's institutions (the European Parliament, Council of Europe, and European Commission) are committed to promoting a model for digital transformation that puts people at the center, is based on European values and EU fundamental rights, reaffirms universal human rights, and benefits all people, businesses, and society as a whole (European Commission, 2022b). Concerning the public sector, the declaration asserts all individuals' rights to access core digital public services in the EU and to participate in the public digital space, safeguarding the right to freedom of expression and information, assembly, and association in the digital environment. The strategic interventions promoted by the EU, including “Next Generation EU” (2021) and the “Pathway to the Digital Decade” (2022), are to be framed in relation to this set of common values.

“Next Generation EU” is a temporary European economic recovery and revitalization tool aimed at restoring the losses caused by the pandemic. The more than 800 billion euros allocated under this tool have been included in the 2021-2027 European budget, with over 26% of the spending dedicated to investment in the digital transition.^1^ The Strategic Program for 2030 “Pathway to the Digital Decade” sets concrete digital goals based on four cardinal points: digital skills, digital infrastructure, the digitization of businesses, and the digitization of public services (European Commission, 2021). The very ambitious objectives of the fourth axis are aimed at ensuring all citizens and businesses can securely access public services online: a 100% online provision of key public services available to European citizens and businesses, 100% of European citizens provided access to medical records (e-records), and 80% of citizens using a form of digital ID. Although there are significant differences in the processes and levels of digitization in different states (Androniceanu et al., 2022), data show that most of the member states that had a lower level of digitization 5 years ago are advancing at a faster pace than the others, thus signaling an overall convergence toward these digitization goals in the EU (European Commission, 2022c).

By allocating the largest share of “Next Generation EU” funds to this area, amounting to 191.5 billion euros, the Italian government launched a massive PA reform and revitalization plan in 2021: under the PNRR plan (Piano Nazionale di Rinascita e Resilienza), 25.1% of the funds in this plan (48 billion) are earmarked for digital transition, with 6.74 billion euros specifically allocated for PA digitization.

Despite improvements, the 2022 data from the Economy and Society Digitization Index (European Commission, 2022c) still places Italy 18th among the 27 EU member states. The most widely used technology for data management and service delivery is the internet, and Italy reached 100% web coverage in the aftermath of the pandemic in all public bodies. The use of social networks for communication between citizens and PA is also widely used (61% of institutions) and spreading rapidly. In contrast, more technologically advanced services are less widespread, with cloud computing use at 44.1%, the use of big data at 7.0%, and the internet of things used by only 6.4%.

Although only 40% of Italian internet users make use of public digital services (compared to an EU average of 65%), this indicator has grown considerably in the last 2 years (with a 10 percentage point increase between 2020 and 2022). Many initiatives have been launched under the PNRR plan, suggesting that this share is likely to increase rapidly. The main programs include the “Digital Italy 2026” plan, the activation of the “Digital PA 2026” platform to monitor the digital implementation in the various PA areas, the “Cloud Italy” strategy for PA digital infrastructure, and the “National Framework for Interoperability.”

At the current stage, e-government platforms that enable digital public engagement (Lupton, 2015; Cava and Penna, 2019) are still relatively uncommon in the Italian context (Visentin, 2018, 2019; Arcidiacono et al., 2021). At the current stage, therefore, this study opted to focus on the use of the basic services offered by the country's PA.

### 1.2. Digital divide and citizens' institutional trust

The implementation of digital public services can bring about increased opportunities and benefits for citizens. In a context of rapid change such as the Italian case, the question is to what extent these benefits are distributed equitably among all citizens and whether they contribute to reducing or amplifying existing inequalities. From the perspective of equitable digital rights development, therefore, it is crucial that the digital divide among citizens be reduced, as the persistence of this divide makes the political potential granted by the network arguable (Van DijK, 2013). The term “digital divide” was initially used to refer to inequalities in access to digital communication technologies for a variety of activities (OECD, 2001), highlighting the link between digital and other forms of inequality (DiMaggio et al., 2001). It was then also used to indicate the role new technologies may play in amplifying inequality (Roth and Luczak-Roesch, 2020, p. 555). Scholars later also included differences in digital skills (Van Dijk, 2012) and how people use the internet under the umbrella of digital divide, recognizing that these differences are in turn intertwined with inequalities (Bentivegna, 2009; Halford and Savage, 2010; Scheerder et al., 2017). Finally, the digital divide has also been discussed with regard to the outcomes achieved through the use of the internet (Scheerder et al., 2017).

The literature has identified multiple factors as related to different forms of digital divide (access, skills, practices and outcomes). In this study, special attention will be given to the factor of education, which will be explored below. The other main ones include belonging to regional areas with less digital infrastructure (Rodríguez-Hevía et al., 2022) and rural areas (Whitacre, 2010), the latter of which are also characterized by fewer opportunities for computing education and training. In Italy, the less-well-equipped macro-areas have historically been those in southern Italy and the islands, although the data do show a significant reduction in the technology gap in recent years (Istat, 2021).

Socioeconomic status is another relevant factor (Hsieh et al., 2008). In terms of ascribed characteristics, age has been found to be an important factor, although there is divergence among surveys as to the significance of this factor (Neves et al., 2018; Mensah and Mi, 2019). Gender is also a factor of digital inequality, as men tend to display greater familiarity with the use of new technologies (Bracciale, 2010; Avveduto, 2019). Since this is a relatively recent phenomenon, research has yet to be conducted in the Italian context systematically analyzing the relationship between these factors (education, area of residence, socio-economic status and gender) and the use of digital public services.

As for the Italian case, it is worth remembering that a low level of using digital public services can be related to a scarce trust in PA and public institutions. So, the development of quality, equitable and inclusive public digital services could help improve the level of institutional trust among citizens. Trust constitutes a structural element of the dynamic functioning of social systems; it is one of the conditions required for their survival (Luhmann, 2002).

Research in Italy has shown widespread distrust in political and administrative institutions among the citizenry for many years. This distrust is shared across different areas of the country and different age groups (Istat, 2016; Cerase, 2018; Mesa and Triani, 2020). A crisis in levels of institutional trust is widespread in many countries, but Italy is especially hard-hit by this phenomenon (Beretta, 2021).^2^ A survey conducted in 2022 shows that, except for law enforcement agencies which enjoy trust levels of 70%, the president of the republic (68%), and schools (56%), all of the country's public institutions fall below the 50% trust threshold, with banks (25%), parliament (23%), and political parties (14%) at the bottom of the list. There is also clearly a critical attitude toward public services: none of the public services surveyed reached a 50% satisfaction level on the part of citizens. Research has shown that the spread of COVID-19 disinformation surrounding health and scientific issues on social networks in Italy has further eroded trust in public institutions and fostered a general state of information crisis (Lovari, 2020). Due to their magnitude and transformative potential in relation to PA services, the reforms currently in progress represent a unique and unrepeatable opportunity to improve the cultural change that can establish a renovated trust and positive relationship between citizens and institutions.

### 1.3. The key role of education

In the complex dynamics of social change, the constraints and opportunities arising from new socio-material configurations are dynamically intertwined with the constraints and opportunities that cultural systems impose on or offer to actors and the way actors cope with new situations (Archer, 1988).

As shown by the literature on the digital divide, in the case of digital transformation, technological equipment and digital infrastructure (at various levels) can inhibit or facilitate access to the internet and digital media, while cultural capital has a greater impact on how ICTs are used and the outcomes of their use (Van DijK, 2013).

From a theoretical point of view, the advantage lies, firstly, in the way that education offers individuals a greater possibility to acquire cognitive tools to deal with the complexity generated by ICT and minimize the impact of the difficulties it may entail (Hsieh et al., 2008). Secondly, the use of ICT and internet activities requires the ability to move non-passively through the vast amount of information available (Bonfadelli, 2002). A higher level of education fosters people's ability to select useful information, evaluate it and manage it effectively (Williams and Dwivedi, 2007; Vicente and Lopez, 2011). People with lower educational levels spend more time online in their free time than those with higher educational levels, but they do so in different ways. They employ digital technologies more frequently for gaming and entertainment activities and less for education, information-seeking, or work-related activities (Van Deursen and van Dijk, 2014).

Referring to the European Commission's assessment of the European Commission (2022), the data indicate that the most significant hurdles to the reform process currently lie mainly in human capital (Italy ranks 25th among the 27 EU countries). In 2022, only 46% of Italians had basic digital skills, compared to the European average of 54%. Similarly, according to Eurostat data it ranks second to last in overall number of tertiary education graduates (17%).

The lack of ICT-skilled staff is also a significant obstacle for both central (55.9%) and local administrations (76.5%).

Studies on Italian youth also show that education remains a strong predictor of both the quality and quantity of digital consumption, as well as cultural consumption tout court (Introini et al., 2020). It is also positively correlated with social participation and institutional trust (Mesa and Triani, 2017).

From this perspective, Italy represents an interesting case study to examine the changes taking place. The country's starting point is characterized by relative structural and cultural backwardness in the level of PA digitization, a low level of digital skills among the population, and a highly bureaucratized, cumbersome, and geographically inconsistent welfare system. On the macro-social level of Europe as a whole, Italy is involved in an accelerated process of digital transformation affecting, on the meso-social level, all spheres of administration–state, regional and local. The impacts of this transformation on the micro-social level have yet to be ascertained in terms of equity as well as citizens' inclusion in service use and trust in the PA.

E-government platforms that enable digital public engagement (Lupton, 2015; Cava and Penna, 2019) are still relatively uncommon in the Italian context (Visentin, 2018, 2019; Arcidiacono et al., 2021). At the current stage, therefore, this study opted to focus on the use of the basic services offered by the country's PA.

### 1.4. Questions and hypotheses

Starting from the theoretical framework of digital rights and the inequalities of opportunity related to the digital divide, this research aims to explore two issues:

I) The impact of different forms of inequality in citizens' propensity to use digital services. Considering the narrowing of the Digital Infrastructure Gap among Italian regions, it is hypothesized that geographical inequalities will have a more attenuated impact while the digital divide related to inequalities in education and social status will prove more persistent.
II) Factors that favorably affect trust and openness to PA digitization. It is hypothesized that the culture factor (level of education, information about ongoing reforms) and experience factor (having benefited from digital services) are the variables most associated with improved institutional trust and citizens' openness to ongoing changes.

This study seeks to make the following key contributions:

I) Considering scenario changes (macro-social level) by highlighting the factors that most affect the conduct of social actors (micro-social level) in order to assess the effectiveness and equity of reform policies (meso-social level).
II) Setting the stage for an initial comparison of the differential use of ICT in state and peripheral government services.
III) Unlike the monitoring systems adopted so far by the Italian government that consider mainly system innovations, this study analyzes changes from the point of view of citizens, considering their practices and degree of informedness, sharing, or resistance toward the reforms that the government is implementing.
IV) Proposing an interpretive lens that treats the cultural dimension of education and training as a strategic factor of activation and inclusion, to be considered pragmatically and programmatically in the implementation of reform processes and in improving trustful PA-citizen relations.

## 2. Materials and methods

The study is based on a web survey commissioned by the Youth Observatory of the Giuseppe Toniolo Institute for Advanced Studies and conducted by Ipsos (a multinational market research and consulting firm) between March 27 and April 7, 2022. The sample, nationally representative of the population of citizens aged 18–64, consisted of 3,002 cases stratified by gender, age group, educational attainment, employment status, and geographical area of residence. The survey was conducted using the Cawi methodology (Computer Assisted Web Interviewing). The online questionnaires were anonymous. No data were collected to identify respondents.

The use of online public services in the past 2 years was surveyed in six strategic sectors: health care (hospital offices/helpdesks); social security and labor (INPS offices/helpdesks; employment centers/employment offices); taxation (revenue agency offices/helpdesks); local government (municipal, provincial and regional offices/helpdesks); chamber of commerce (industry and crafts offices/helpdesks); and schools and universities (offices/helpdesks).

Citizens' attitudes toward PA were measured with the following indicators: the degree of current trust in PA; the perceived need for change; the respondent's degree of knowledge about the PNRR reform plan; confidence in the implementation of reforms; expectations about the improvements brought by digital services; perceptions of the problems that the ongoing digital transition may bring.

The above variables were cross-referenced with respondents' levels of education, operationalized in three modes: primary and low secondary education (ISCED 1-2); upper secondary education (ISCED 3); and tertiary education (ISCED 4-5-6-7).

To profile the users who make use of e-government services and those who have a positive attitude toward the digital transformation of public services, a binary logistic regression model was used.

## 3. Results

### 3.1. The use of public digital services

Data about the use of public services show some significant differences between the different sectors analyzed (Table 1).

**Table 1:** Citizens who have interacted with public services in the past 2 years (percentages).

	**Healthcare**	**Local adm**.	**Soc. sec. and labor**	**Taxation**	**School and univ**.	**Chamber of comm**.
Used services	84.0	75.1	65.5	50.6	46.8	28.5
Did not need to use them	16.0	24.9	34.5	49.4	53.2	71.5

N = 3,002

The sectors with the highest utilization rates are healthcare and local government. During the past two years, 84.0 and 75.1% of respondents, respectively, have made use of them. These services are essential systems of societal protection that offer multiple benefits to the general population. At the intermediate level are social security and labor (65.5% of users), taxation (50.6%) and the school and university field (46.8%), which are used by specific but fairly large segments of the population. This intermediate use-level is followed by the chamber of commerce, which has a smaller target audience (28.5%) consisting of entrepreneurs, traders and artisans.

There are significant differences among the sectors considered here in terms of how they interact with citizens (Table 2). Those with the highest rate of online interactions are tax offices (46.0%), followed by welfare and labor offices (44.5%). These services pertain mainly to the central state administration, the component of the country's PA that began the digitization process earliest and is this furthest advanced. On the opposite end are health services and local government, which show 14.6% and 21.2% of online interactions, respectively. Rather than belonging directly to the central government, these services are run by peripheral entities (regions, health districts, municipalities) whose levels of digitization are currently uneven (Istat, 2021; European Commission, 2022c). They also offer mostly care and outreach services that often involve direct, face-to-face contact with citizens. For these reasons, the most frequent modes of interaction are those taking place “in person” followed secondarily by interactions “both online and in person.” The same reasoning applies to schools, universities and chambers of commerce, although these latter are characterized by a significantly higher level of online interactions. Schools and universities in particular have undergone a process of forced digitization since the outbreak of the COVID-19 pandemic (Colombo et al., 2022; Mesa, 2022). Chamber of Commerce services mostly involve bureaucratic procedures and are therefore more easily digitized.

**Table 2:** Ways in which citizens approached public services (percentages).

	**Healthcare**	**Local adm**.	**Soc. sec. and labor**	**Taxation**	**School and univ**.	**Chamber of comm**.
Online	14.6	21.2	44.5	46.0	29.5	27.3
Both online and in person	38.2	31.1	29.2	29.2	39.1	31.2
In person	47.2	47.7	26.4	24.8	31.4	41.5
	N = 2,521	N = 2,255	N = 1,966	N = 1,519	N = 1,406	N = 856

Overall, considering transactions carried out both partially and fully online, three out of four citizens can be said to have interacted digitally with social security and labor (73.6%) and tax offices (75.2%), followed by school and university (68.6%) and chamber of commerce (58.5%) services. Digital users account for just over half of interactions for health services (52.8%) and local government (52.3%).

Table 3 compares the shares of PA platform beneficiaries by educational attainment. With the exception of schools and universities, in all other areas there is a statistically significant relationship between high educational level and use of PA platforms. Among respondents with tertiary education, the share of digital users is between 61.1% (health services) and 81.3% (social security and labor). For those with lower levels of education, the share ranges from 43.5% (local government) to 69.2% (taxation). The minimum gap between graduate and low-educated PA platforms beneficiaries is 6.9 percentage points, in the case of schools and universities. The maximum one is 19.2 points, in the case of local governments.

**Table 3:** Share of PA platform beneficiaries by educational attainment (percentages).

	**Health care**	**Local adm**.	**Soc. sec. and labor**	**Taxation**	**School and univ**.	**Chamber of comm**.
Tertiary education (ISCED 4-5-6-7)	61.1	62.7	81.3	79.5	61.4	76.2
Upper secondary education (ISCED 3)	56.3	56.2	75.2	78.5	60.5	72.8
Primary and lower secondary education (ISCED 1-2)	46.2	43.5	68.7	69.2	54.5	58.9
*N* =	2,521	2,255	1,966	1,519	1,406	856

### 3.2. Trust in PA and expectations regarding the digital transformation

The data concerning citizens' degree of trust in Italy's PA and ongoing digital transformation show a significant polarization of positions (Table 4). Overall, only slightly more than half of the respondents (54.6%) expressed a positive level of trust toward PA, with a rating between 6 and 10. This data is related in part to the way citizens relate to PA. Among PA platform laggers (those who have never approached public services digitally) trust drops to 46.4%, while among PA platform beneficiaries it is 57.1%. Considering educational attainment levels, highly educated respondents report a much higher level of trust, reaching 63.4%.

**Table 4:** Opinions about PA by mode public service use and educational attainment (percentages).

	**%**	**Use of PA platforms**		**Education level**			**Total**
		**Laggers**	**Beneficiaries**	**Low**	**Medium**	**High**	
Trust in public administration^*^	Low (val. 1–5)	53.6	42.9	48.7	45.7	36.6	45.4
	High (val. 6–10)	46.4	57.1	51.3	54.3	63.4	54.6
Change in trust in PA after the pandemic^*^	Increased	7.4	12.8	10.0	10.1	17.9	11.4
	Unchanged	60.2	55.6	55.6	57.8	57.3	56.8
	Decreased	32.4	31.6	34.4	32.1	24.8	31.8
Is aware of the PA reforms contained in the PNRR plan^*^	Yes	16.5	30.8	20.5	27.9	44.2	27.5
	No	54.9	48.0	52.2	50.8	40.1	49.5
	Don't know	28.6	21.2	27.3	21.3	15.7	23.0
Degree of confidence in reform implement^*^	Low (val. 1–5)	56.5	45.8	53.9	46.4	37.8	48.3
	High (val. 6–10)	43.5	54.2	46.1	53.6	62.2	51.7
Digitization of PA^*^	Will improve the functioning of PA	59.1	70.6	61.1	71.4	77.8	67.9
	Will create more difficulties	40.9	29.4	38.9	28.6	22.2	32.1
Reasons why digitization may create difficulties^**^	The lack of attention to users	24.0	23.9	23.4	25.6	22.5	23.9
	Users' poor digital skills	54.8	54.9	57.2	53.0	49.2	54.9
	The risk of privacy violation	15.3	15.3	14.1	15.3	20.0	15.3
	Other reason...	5.9	5.9	5.3	6.1	8.3	5.9

^*^N = 3,002; ^**^N = 965.

For the majority of citizens (56.8%), their level of trust in PA remained unchanged after the pandemic. Of those whose opinion changed, more respondents reported a worsening level (31.8%) than an improvement in trust (11.4%). This trend is similar between PA platform laggers and beneficiaries, and was not found to change significantly after the pandemic outbreak even when considering educational attainment.

The survey shows that slightly more than one in four respondents (27.5%) are aware of the PA digitization reforms planned as part of the PNRR plan. The difference in knowledge level between PA platform laggers and beneficiaries is more than 14 percentage points in favor of beneficiaries; between low-educated and highly educated citizens, the gap is nearly 24 percentage points.

Data on expectations for the implementation of PA reforms are similar to those expressed more generally regarding trust in PA. In this case as well, citizens are fairly evenly divided between skeptical (48.3%) and trusting (51.7%). There is a significantly higher level of trust among PA platform beneficiaries (54.2%) than among laggers (43.5%) as well as among college graduates (62.2%) as opposed to those with lower levels of education (46.1%).

Although doubts about the viability of the reforms are widespread, 67.9% of respondents say they believe digitization will improve the quality of services. The share of optimists among PA platform laggers is 59.1% and rises to 70.6% among beneficiaries. Among citizens with low education and high education, the share is 61.1 and 77.8%, respectively.

Overall, 32.1% of the sample believe that the difficulties brought by digitization will outweigh the benefits. The first cause of these difficulties is identified as the low level of users' digital skills (54.9%), followed by a lack of attention granted to users by public services (23.9%) as a result of the automation of procedures and the loss of direct contact with service providers. Less concern is expressed about the risks of privacy violations related to the digital management of sensitive user data (15.3%). Regarding this concern, the different profiles of users investigated here show substantial agreement.

### 3.3. Factors related to the use of digital services and trust in PA

To estimate the effects of a set of factors on digital services use and trust in PA, two logistic regression analyses were performed. This type of analysis focuses on defining and predicting a dichotomous outcome centered on a set of independent variables (Gallucci and Leone, 2012).

The explanatory variables considered pertain to the following dimensions (Table 5): individual ascribed characteristics (age; gender); acquired characteristics (study/job/unemployment status; educational attainment); and characteristics of the area in which respondents live (macro-areas; degree of urbanization; number of inhabitants). Some subjective characteristics (emotional and psychological wellbeing; physical wellbeing; knowledge of PA reform; expectations about digital public services) were also considered as intervening variables.

**Table 5:** Variables included in logistic regression.

**Independent variables**	**%**
**Age**	
18–24 years (reference)	11.4
25–34 years	17.6
35–44 years	20.8
45–54 years	26.4
55–64 years	23.8
**Gender**	
Male (reference)	49.9
Female	50.1
**Main activity**	
Worker (reference)	58.1
Student	8.0
Housewife	13.2
Unemployed/receiving redundancy pay	11.5
Other condition	9.2
**Educational attainment**	
Tertiary education (ISCED 4-5-6-7) (reference)	17.8
Upper secondary education (ISCED 3)	36.7
Primary and Low secondary education (ISCED 1-2)	45.5
**Macro-areas**	
North-east (reference)	19.4
North-west	26.5
Center	19.7
South and islands	34.4
**Degree of urbanization**	
Cities (reference)	38.8
Towns and suburbs	46.5
Rural areas	14.7
**Number of inhabitant**	
Up to 10,000 inhabitants (reference)	26.9
10,000 to 30,000 inhabitants	24.2
30,000 to 100,000 inhabitants	22.7
100,000 to 250,000 inhabitants	8.7
More than 250,000 inhabitants	17.6
**Physical wellbeing**	
Low level of wellbeing (val. 1-5) (reference)	22.6
High level of wellbeing (val. 6-10)	77.4
**Emotional and psychological wellbeing**	
Low level of wellbeing (val. 1–5) (reference)	27.4
High level of wellbeing (val. 6–10)	72.6
**Knowledge of PA reform**	
No (reference)	
Yes	
**Expectations regarding digital public services**	
They will make PA worse (reference)	67.9
They will improve PA	32.1

N = 3,002.

Table 6 presents the results of the regression performed on the dependent variable “use of digital public services” (0 = no online interaction with public services; 1 = at least one online experience with public services). Data from Model 1 show that, excluding estimated effects on the other variables, age, and gender do not significantly affect the probability of having had at least one online interaction with PA in the past 2 years. Occupation, on the other hand, has a significant effect. Working status increases the probability of using online services and student status does not affect this probability while all other conditions reduce the odds of using online services. The most significant variable is educational attainment. The most penalized category is citizens with primary and low secondary education. Regarding the characteristics of the area in which respondents live, geographical area and population size were not found to have any significant effects whereas citizens living in rural areas are penalized.

**Table 6:** Results of logistic regression models of digital public service use (models 1 and 2).

**Independent variables**	**Use of digital public services model 1**	**Use of digital public services model 2**
**Age**		
18–24 years (reference)		
25–34 years	0.091	0.145
35–44 years	−0.116	−0.042
45–54 years	0.029	0.124
55–64 years	−0.203	−0.151
**Gender**		
Male (reference)		
Female	−0.094	−0.040
**Main activity**		
Worker (reference)		
Student	−0.120	−0.070
Housewife	−0.624^***^	−0.630^***^
Unemployed/receiving redundancy pay	−0.717^***^	−0.666^***^
Other condition	−0.552^***^	−0.566^***^
**Educational attainment**		
Tertiary education (ISCED 4-5-6-7) (reference)		
Upper secondary education (ISCED 3)	−0.518^**^	−0.429
Primary and Low secondary education (ISCED 1-2)	−1.254^***^	−1.110^***^
**Territorial areas**		
North-east (reference)		
North-west	−0.031	−0.051
Center	−0.248	−0.274
South and islands	−0.156	−0.196
**Degree of urbanization**		
Cities (reference)		
Towns and suburbs	−0.267	−0.259
Rural areas	−0.714^**^	−0.735^**^
**Number of inhabitant**		
Up to 10,000 inhabitants (reference)		
10,000 to 30,000 inhabitants	0.185	−0.174
30,000 to 100,000 inhabitants	−0.010	−0.270
100,000 to 250,000 inhabitants	−0.111	−0.367
More than 250,000 inhabitants	−0.155	−0.167
**Physical wellbeing**		
Low level of wellbeing (val. 1–5) (reference)		
High level of wellbeing (val. 6–10)		0.111
**Emotional and psychological wellbeing**		
Low level of wellbeing (val. 1–5) (reference)		
High level of wellbeing (val. 6–10)		−0.377^**^
**Knowledge of PA reform**		
No (reference)		
Yes		0.616^***^
**Expectations regarding digital public services**		
They will make PA worse (reference)		
They will improve PA		0.343^***^
**Trust in PA**		
Low level of trust (val. 1–5) (reference)		
High level of trust (val. 6–10)		0.302^**^

^***^p < 0.001, ^**^p < 0.005, ^*^p < 0.01.

In Model 2, in addition to the variables shown in Table 5, trust in PA was also included as an intervening variable. Analysis of the intervening variables shows that psycho-emotional wellbeing has no significant effect, while having physical problems increases the likelihood of using online services. The use of online services is positively correlated with knowledge of PA reforms, positive expectations regarding the changes taking place, and trust in PA. The introduction of intervening variables in the regression model slightly reduces the negative impact of low educational attainment and unemployment status.

Table 7 presents the results of the regression performed on the dependent variable “trust in PA” (0 = low trust; 1 = high trust). Model 3, with only two variables, shows significant relationships. With respect to the employment status variable, unemployed/receiving redundancy pay contributes to reduced levels of trust. Concerning the education variable, the data show a negative effect for citizens with middle and low levels of education.

**Table 7:** Results of logistic regression models of trust in PA (models 3 and 4).

**Independent variables**	**Trust in PA model 3**	**Trust in PA model 4**
**Age**		
18–24 years (reference)		
25–34 years	−0.375	−0.362
35–44 years	−0.261	−0.225
45–54 years	−0.185	−0.169
55–64 years	−0.149	−0.175
**Gender**		
Male (reference)		
Female	0.059	0.188
**Main activity**		
Worker (reference)		
Student	−0.135	−0.066
Housewife	0.109	0.173
Unemployed/receiving redundancy pay	−0.670^***^	−0.502^***^
Other condition	−0.125	−0.054
**Educational attainment**		
Tertiary education (ISCED 4-5-6-7) (reference)		
Upper secondary education (ISCED 3)	−0.403^***^	−0.319^**^
Primary and Low secondary education (ISCED 1-2)	−0.516^***^	−0.288
**Territorial areas**		
North-east (reference)		
North-west	−0.041	−0.028
Center	−0.068	−0.075
South and islands	−0.264	−0.270
**Degree of urbanization**		
Cities (reference)		
Towns and suburbs	0.137	0.195
Rural areas	0.465	0.594^**^
**Number of inhabitant**		
Up to 10,000 inhabitants (reference)		
10,000 to 30,000 inhabitants	0.186	0.198
30,000 to 100,000 inhabitants	0.276	0.346
100,000 to 250,000 inhabitants	0.255	0.336
More than 250,000 inhabitants	0.215	0.285
**Physical wellbeing**		
Low level of wellbeing (val. 1–5) (reference)		
High level of wellbeing (val. 6–10)		0.683^***^
**Emotional and psychological wellbeing**		
Low level of wellbeing (val. 1–5) (reference)		
High level of wellbeing (val. 6–10)		0.143
**Knowledge of PA reform**		
No (reference)		
Yes		0.560^***^
**Expectations regarding digital public services**		
They will make PA worse (reference)		
They will improve PA		0.429^***^
**Use of digital public services**		
Never used (reference)		0.300^**^
Used at least once		

^***^p < 0.001, ^**^p < 0.005, ^*^p < 0.01.

In Model 4, in addition to the variables shown in Table 5, the use of digital public services was also included as an intervening variable. The results show a positive association between trust and psychological-emotional wellbeing, knowledge of current reforms, and positive expectations about digital public services. The use of online services also increases the level of trust in PA. The introduction of the intervening variables in the regression model eliminates the negative impact exerted by low educational attainment and mitigates the impact exerted by unemployment status while showing a positive relationship between trust in PA and rural area membership.

## 4. Discussion

The goal of this study was to ascertain how educational inequalities affect the relationship between Italian citizens and PA in this phase of the digital transition.

The results confirm that significant differences exist among different PA sectors. Considering the most frequent mode of interaction, services can be seen as falling into one of three categories: (1) centralized services with high digitization (social security and labor; taxation) in which the most popular mode of interaction is online; (2) decentralized services with medium digitization (schools and universities) in which the mixed form of in-person and online interaction prevails; (3) decentralized services with low digitization (health care; local government; chamber of commerce) in which in-person contact is still the most popular mode.

Regarding the first issue considered, the hypothesis of education as the main predictor of digital services use is confirmed. This incidence is significant in five out of the six areas surveyed. With respect to social status and ascribed variables, the incidence of working status is confirmed while age and gender variables are found to have zero affect. The hypothesis of an attenuation in the effect of macro-area differences is also confirmed, while use differences among citizens in rural vs. urban areas remain significant. The data indicate that the development of digital services is patchy, with this unevenness following a complex geographical configuration that does not simply correspond to the country's traditional north-central-south divide.

Coming to the second question, the hypothesis that education and experience of using digital services have positive influence on trust in PA is confirmed.

The data regarding trust and expectations for change confirm that there is intense skepticism among citizens. The level of trust seems to have become even lower after COVID-19 for just under a third of the sample. This underlying attitude also extends to the weak confidence citizens express as to whether the country's PA can really improve. This mood is also fueled by a lack of knowledge about the investments and measures that are being implemented.

All of these aspects were found to be significantly related to both educational attainment and having past experience of online interaction with PA.

Overall, the data provide a snapshot of Italy's “work in progress” situation in terms of the digital transition of PA, cultural acceptance of the paradigm shift, and citizens' embracing of smarter ways of relating to public services.

It can be considered a good result the fact that three out of four citizens have interacted online at least once with a public service in the past 2 years (2020–2022). But there is still a long way to go to achieve the EU-established goal of 100% of citizens having access to all essential digital services by 2030.

In this middle ground between old and new PA reconfigurations, the data show both positive advances and critical challenges with respect to the goal of ensuring equal rights and opportunities for all. The digital divide between macro-areas is narrowing, as is the generational and gender gap. The ability to access services electronically is greater among citizens who have physical impediments and difficulty traveling to PA offices. However, some social categories are in danger of being left behind: primarily, citizens with fewer cultural resources, those excluded from the education circuit and labor market (especially housewives, the unemployed), and citizens living in rural areas. These categories would instead need to receive more support from welfare systems. Human capital formation is thus a strategic factor and a key challenge to ensure substantive, universal access to and participation in digital services, as well as participation in the “digital public sphere” that is gradually taking shape.

Alongside structural measures targeting technological infrastructure and service development, the country needs to formulate a strategic plan for digital culture and skills training, especially among the most at-risk groups. Given the low level of knowledge, the plan should start by building a common information base. A wide-ranging campaign to raise awareness about the reforms taking place and their concrete benefits could foster a more collaborative and open attitude on the part of citizens. Second, such a campaign should include a diverse set of initiatives aimed at enabling citizens to use services.

Citizens' major points of resistance to PA digitization are cultural, and stem from the attachment to the traditional “proximity approach” to public institutions, from which derives the fear of not being able to use new procedures and not having personal control when interacting with services (an echo of the Banfield's “Italian familism” can be tracked here, cfr. Banfield, 1958). Therefore, in local services that continue to involve intense direct contact with beneficiaries, facilitators could be provided to support citizens in setting up digital user accounts and managing procedures and contingencies. With this in mind, for example, some provinces and regions are experimenting with opening digital facilitation centers using PNRR funds. These are “physical access points, usually located in libraries, schools and community centers, that provide citizens with both in-person and online digital skills training in order to support digital inclusion.”^3^

The results of this exploratory study also point to some possible avenues for further study in education research:

- With respect to the diffusion of digital public services: it would be advisable to develop a systematic monitoring of the impact of educational inequalities and other forms of inequality connected to the digital divide over time; the status of senior citizens, a group not included in this research and mostly low educated, should also be investigated, as they represent a further population at risk of exclusion;
- To improve citizens' digital competences, in particular those of the more vulnerable groups: it would be advisable to map and study the various initiatives (policy measures, projects, services) aimed at actively supporting citizens in their use of digital public services and to further develop the practices (training courses, tutoring activities, peer to peer), methods (online, face-to-face, mixed) and actors involved (administrations, third-sector actors, social networks, committees);
- To reduce misinformation related to public services and improve levels of institutional trust, it is important to observe the development of unprecedented forms of communication between citizens and PA through social media, both media networks related to citizen services and those related to public utility issues;
- With respect to the youth population, the training programs and initiatives available as part of formal/non-formal/informal educational pathways (particularly those associated with civic education curricula (Losito, 2021; Mesa, 2023) aimed at fostering digital skills and citizens' knowledge of their rights and responsibilities should be monitored specifically in relation to the use of ICT and the internet in the public sphere.

## Data availability statement

The datasets presented in this article are not readily available because the first version of the survey dataset was released in Spring of 2022 and at moment it is available only for members of the Giuseppe Toniolo Institute for Advanced Studies. Requests to access the datasets should be directed to diego.mesa@unicatt.it.

## Author contributions

DM had substantial contributions to the design, drafting, revision, acquisition, interpretation, and final approval of the data and work.

## Footnotes

^1^Source: European commission website https://commission.europa.eu/business-economy-euro/economic-recovery/recovery-and-resilience-facility_en consulted on 12/29/2022.

^2^Source: https://www.worldvaluessurvey.org/wvs.jsp consulted on 12/29/2022.

^3^ https://padigitale2026.gov.it/misure/
